# Percutaneous cryoablation for clinical T3a renal cell carcinoma (< 7 cm) with segmental vein involvement or perinephric fat invasion based on preoperative evaluation of high-resolution multidetector computed tomography scan

**DOI:** 10.1007/s11604-022-01297-8

**Published:** 2022-06-21

**Authors:** Mayu Uka, Toshihiro Iguchi, Nanako Okawa, Yusuke Matsui, Koji Tomita, Noriyuki Umakoshi, Kazuaki Munetomo, Hideo Gobara, Motoo Araki, Takao Hiraki

**Affiliations:** 1 Department of Radiology, Dentistry and Pharmaceutical Science, Okayama University Graduate School of Medicine, 2-5-1 Shikata-cho kita-ku, Okayama, 700-8558 Japan; 2 Department of Radiological Technology, Graduate School of Health Sciences, Okayama University, 2-5-1 Shikata-cho kita-ku, Okayama, 700-8558 Japan; 3 Division of Medical Informatics, Okayama University Hospital, 2-5-1 Shikatacho, Kitaku, Okayama, 700-8558 Japan; 4 Department of Urology, Dentistry and Pharmaceutical Science, Okayama University Graduate School of Medicine, 2-5-1 Shikata-cho kita-ku, Okayama, 700-8558 Japan

**Keywords:** Kidney neoplasms, Cryosurgery, Image-guided

## Abstract

**Purpose:**

To retrospectively assess the feasibility, safety, renal function, technique efficacy rate, and survival of patients with clinical T3a renal cell carcinoma (RCC).

**Materials and methods:**

Sixteen cryoablation sessions were performed in 14 patients (10 men; mean age, 69.8 ± 10.5 years; range, 49–90 years) with 14 clear cell T3a RCCs (mean, 3.3 ± 0.9 cm; range, 1.9–5.2 cm). One patient was on dialysis. Transcatheter arterial embolization was performed before cryoablation in 15 sessions. The primary endpoint was the technique efficacy rate. The secondary endpoints included feasibility, safety, renal function, and survival.

**Results:**

Cryoablation was technically successful in all RCC cases. In two RCCs, cryoablation was performed twice because of local tumor progression. No major adverse events were observed. All patients were alive without metastases, with a median follow-up of 45 months (6−93 months). Complete response was achieved by cryoablation in 11 RCCs (78.6%). The primary and secondary technique efficacy rates were 77.1% and 84.4% at 1 year, 57.9% and 73.9% at 3 years, and 57.9% and 73.9% at 5 years, respectively. One patient underwent dialysis given a total contralateral nephrectomy due to another RCC 1 month after initial cryoablation and a total ipsilateral nephrectomy 46 months after initial cryoablation due to local progression. Except for two dialysis patients, of the 12 patients with a median follow-up of 41 months (6–93 months), none were on dialysis.

**Conclusion:**

Cryoablation was safe and effective in T3a RCC, which mainly involved the renal venous branches and may represent an alternative treatment for inoperable patients.

## Introduction

The incidence of renal cell carcinoma (RCC) is increasing in light of the widespread use of diagnostic imaging modalities, such as computed tomography (CT), ultrasound, and magnetic resonance imaging (MRI) [1]. Most incidental RCCs are small in size and localized in the kidney (i.e., T1a RCC), but they are sometimes found at an advanced stage (e.g., ≥ 7 cm RCC, locally advanced RCC, or RCC with distant metastasis). The standard of care for stage I and II patients (i.e., T1 and T2 RCC without distant metastasis) is surgical resection. Similarly, the gold standard for the treatment of locally advanced RCC (i.e., T3a RCC) is radical nephrectomy in patients without distant metastasis [2].

Recently, percutaneous thermal ablation, including cryoablation, radiofrequency ablation, and microwave ablation, has been widely used as an alternative curative therapy, mainly for inoperable patients with small RCC. Their therapeutic efficacies are excellent [3]. In particular, cryoablation is considered safe and effective because the ablation zone (called “ice ball”) may be confirmed in real-time during treatment. Therefore, this therapy is sometimes performed for patients with more advanced RCC (i.e., T1b and T2 RCCs) as well as those with T1a RCC [4–6]. In addition, as a challenging small case series, Atwell et al. reported the results of percutaneous cryoablation for T3a RCC with sufficient local control and safety [7].

In inoperable patients, few treatment options for T3a RCC are available. Unfortunately, some patients cannot undergo surgery under general anesthesia for various reasons, such as advanced age, poor performance status, organ malfunctions, and other medical comorbidities. Selected patients do not prefer surgical treatment, considering the possibility of chronic dialysis following radical or partial nephrectomy. We speculate that percutaneous cryoablation may provide some local control for locally advanced RCC without metastasis (i.e., T3a, N0, and M0 stage). If this therapy is safe and effective for patients with T3a RCC, the treatment strategy for such patients will be expanded. This study sought to retrospectively assess the feasibility, safety, local tumor control, change in renal function, and oncologic outcomes of cryoablation in patients with clinical T3a RCC.

## Materials and methods

This retrospective study was approved by our institutional review board (approval number, KEN2102-015) and waived the requirement for obtaining informed consent to use the patients’ medical data. Written informed consent was obtained from all patients before treatment.

### Patient and tumors

Between May 2012 and March 2020, 530 cryoablation procedures were performed percutaneously for 545 renal tumors in 429 patients at our institution. Clinically, 523 tumors were diagnosed with renal cancer; T1a: 484 tumors, T1b: 22 tumors, T2: two tumors, and T3: 15 tumors. The inclusion criteria were as follows: (i) histologically proven RCC and (ii) clinical T3a RCC diagnosed by preprocedural dynamic contrast-enhanced CT (CECT). The exclusion criteria were as follows: (i) lymph node or distant metastasis diagnosed by preprocedural CECT, ii) no dynamic CECT evaluation before cryoablation, or (iii) < 6 months of follow-up.

In our institution, nephrectomy is basically performed for patients with tumors diagnosed as T3a on pretreatment imaging according to standard treatment guidelines.

Our clinical indications for renal cryoablation included contraindications to surgery, major comorbidities, and patients’ willingness to consider procedural tolerance. The cases in all patients with T3a N0 M0 RCC were discussed at a multidisciplinary radiology team conference and discussion with a urologist to probe the feasibility of treatment, with advantages and disadvantages, weighed against the alternative of radical nephrectomy before treatment.

### Study endpoint

The primary endpoint was the technique efficacy rate of cryoablation for clinical T3a RCC. The secondary endpoints were feasibility (i.e., technical success), safety, renal function, and survival. The primary technique efficacy rate was defined as the percentage of target RCCs successfully eradicated following the initial procedure. The secondary technique efficacy rate was defined as including tumors that had undergone successful repeat ablation following the identification of local tumor progression [8]. Technical success was defined as the completion of cryoablation according to the procedure protocol [8]. Adverse events (AEs) that occurred within 30 days of the cryoablation procedure were assessed according to the Clavien-Dindo classification [9]. Major AEs were defined as Clavien-Dindo classification ≥ grade III.

### Imaging evaluation before cryoablation

Prior to cryoablation, dynamic CECT images with 1-mm or 1.25-mm slice thickness were obtained before and after the intravenous administration of 300 mgI/ml contrast medium, at a dose of 2.0 g iodine per kg of weight and a fixed injection duration of 30 s during the corticomedullary (36 s delay), nephrogenic (53 s delay), and excretory (240 s delay) phases. In the context of this study, dynamic CECT (axial, coronal, and sagittal images) of all 545 renal tumors before cryoablation was reviewed by consensus by two board-certified diagnostic radiologists (M.U. and N.O.) with 16 and 12 years of experience, respectively. A clinical T3a RCC was defined as the following findings: (i) tumor extending to the renal vein or its segmental branches, (ii) tumor invading the pelvicalyceal system, (iii) tumor invading perirenal fat, or (iv) tumor invading renal sinus fat (i.e., peripelvic fat) but not beyond Gerota fascia [10]. When the intravenous extension was diagnosed, the level of extension, the length of the intravenous extension, and the borderline of the lesion (clear or indistinct) were evaluated.

### Transcatheter arterial embolization (TAE) prior to cryoablation

Prior to cryoablation, TAE was performed to improve visualization of the target RCC during CT-fluoroscopy-guided procedures and to reduce the heat sink effect and the potential risks of bleeding and dissemination caused by cryoprobe insertion. Super selective intra-arterial embolization of the feeding artery to the tumor was performed using a 7:3 mixture of absolute ethanol and iodized oil [11]. Additional coil embolization was performed when there was a large arterial branch in the planned puncture route and/or when prior marking of the extension part of the RCC to the renal sinus was preferred.

### Cryoablation procedure

Cryoablation was performed percutaneously for prone-positioned inpatients under CT-fluoroscopy (Aquilion 64; Cannon Medical Systems, Otawara, Japan) guidance by six experienced interventional radiologists (M.U., K.T., T.I., H.G., T.H., and others) in the interventional radiology room. All procedures were performed under local anesthesia with conscious sedation using an intravenous infusion of fentanyl and hydroxyzine using an argon- and helium-based cryoablation system (CryoHit, Galil Medical, Yokneam, Israel) with 17-gauge cryoprobes (Ice-Rod or Ice-Seed, Galil Medical). The type and number of cryoprobes used and the array of cryoprobes inserted in the targets were assessed by experienced interventional radiologists based on their consensus. When possible, operators directly inserted a cryoprobe into the extension of the RCC to the renal sinus. When this was impossible, operators inserted it as close as possible to the site of venous invasion.

The ablation protocol included two 10- to 15-min freezing cycles, separated by two or more minutes of passive thawing, after placing 3−5 cryoprobes at the target site. CT scan was performed to assess the ablation zone (i.e., the ice ball) immediately after freezing. The ablation sought to cover the target RCC with an adequate (≥ 6 mm from tumor margin) circumferential ablation margin [12]. If the ablation zone was not sufficient to treat the entire tumor, additional freeze and thaw cycles were performed after repositioning the cryoprobes.

Saline with a 2% contrast medium was infused to displace a non-target organ (e.g., the colon) away from the expected ablation zone (i.e., hydrodissection) when the RCC was adjacent to the non-target organ.

### Follow-up

Dynamic CECT was performed to assess therapeutic efficacy, complications, and distant metastasis at 1, 3, and 6 months after cryoablation and at 6−12 month intervals thereafter. Since the patients were also evaluated using dynamic CT preoperatively, they were followed up using dynamic CT after treatment for accurate comparative evaluation. In the ablated kidney, axial and coronal images of each phase were reconstructed with a 5 mm slice thickness. The appearance of a nodular focus exhibiting contrast enhancement within or adjacent to the ablation zone indicates local tumor progression [8]. In patients with renal dysfunction (estimated glomerular filtration rate [eGFR] < 30 mL/min), a non-enhanced MRI was performed. On non-enhanced MRI, local tumor progression was defined as a new and enlarging focus of hyperintensity within or adjacent to low signal intensity in the ablated area on T2-weighted images [13].

### Statistical analysis

The primary and secondary technique efficacy rates were assessed using the Kaplan–Meier method. To evaluate renal function, eGFR at 6 months after cryoablation was compared with the baseline value (i.e., eGFR prior to treatment) using the paired *t* test. The length of the intravenous extension in the local progression and non-local progression groups after one cryoablation session was compared using the Mann − Whitney *U* test. Statistical significance was set at *p* < 0.05. All statistical analyses were performed using SPSS software, version 26 (IBM).

## Results

Fourteen patients (10 men and four women; mean age, 69.8 ± 10.5 years; median age, 71 years; range, 49–90 years) with histologically proven 14 clear cell RCCs (mean, 3.3 ± 0.9 cm; median, 3.0 cm; range, 1.9–5.2 cm) were included in this study. Eight RCCs were located in the right kidney and six in the left kidney. One patient had a solitary kidney. Two of the 14 patients had recurrent RCCs in the region of prior partial nephrectomy or radiofrequency ablation. Ten of the 14 RCCs invaded the segmental vein, two invaded the pelvicalyceal system, one invaded the perinephric fat, and one invaded both the segmental vein and perinephric fat. The 11 cases with an intravenous extension all extended to the distal one-third of the segmental vein, and the mean length of the intravenous extension was 9.1 ± 2.3 mm (5−12 mm). The border of the intravenous extension was clear in nine cases and indistinct in two cases. One patient was on dialysis. The mean eGFR value prior to treatment was 57.5 ± 23.1 mL/min (6.4–88.3 mL/min). The patient and tumor characteristics are presented in Table 1.

**Table 1:** Characteristics of 14 patients, 14 T3a RCCs, and 16 procedures

Variable		Value
Patient characteristics (*n*=14)		
Age (y)	Mean ± SD (Range)	69.8 ± 10.5 (49-90)
Gender	Male/Female	10/4
Kidney	1/2	1/13
eGFR (mL/min/1.73m^2^) before treatment	Mean ± SD (Range)	57.5 ± 23.1 (6.4-88.3)
RCC characteristics		
Size (cm)	Mean ± SD (Range)	3.3 ± 0.9 (1.9-5.2)
Laterality	Right/Left	8/6
Subtype of RCC	Clear cell RCC	14
Site of invasion	Segmental vein/Pelvicayceal system/Perinephric fat/Both segmental vein and perinephric fat	10/2/1/1
Procedural characteristics (*n* = 16)		
Prior transacatheter arterial embolization	Yes/No	15/1
Number of cryoprobes	Mean ± SD (Range)	3.1 ± 0.5 (2-5)
Total ablation time (min)	Mean ± SD (Range)	51.6 ± 21.9 (30-105)

*RCC* renal cell carcinoma; *SD* standard deviation; *eGFR* estimated glomerular filtration rate

**Fig. 1:**
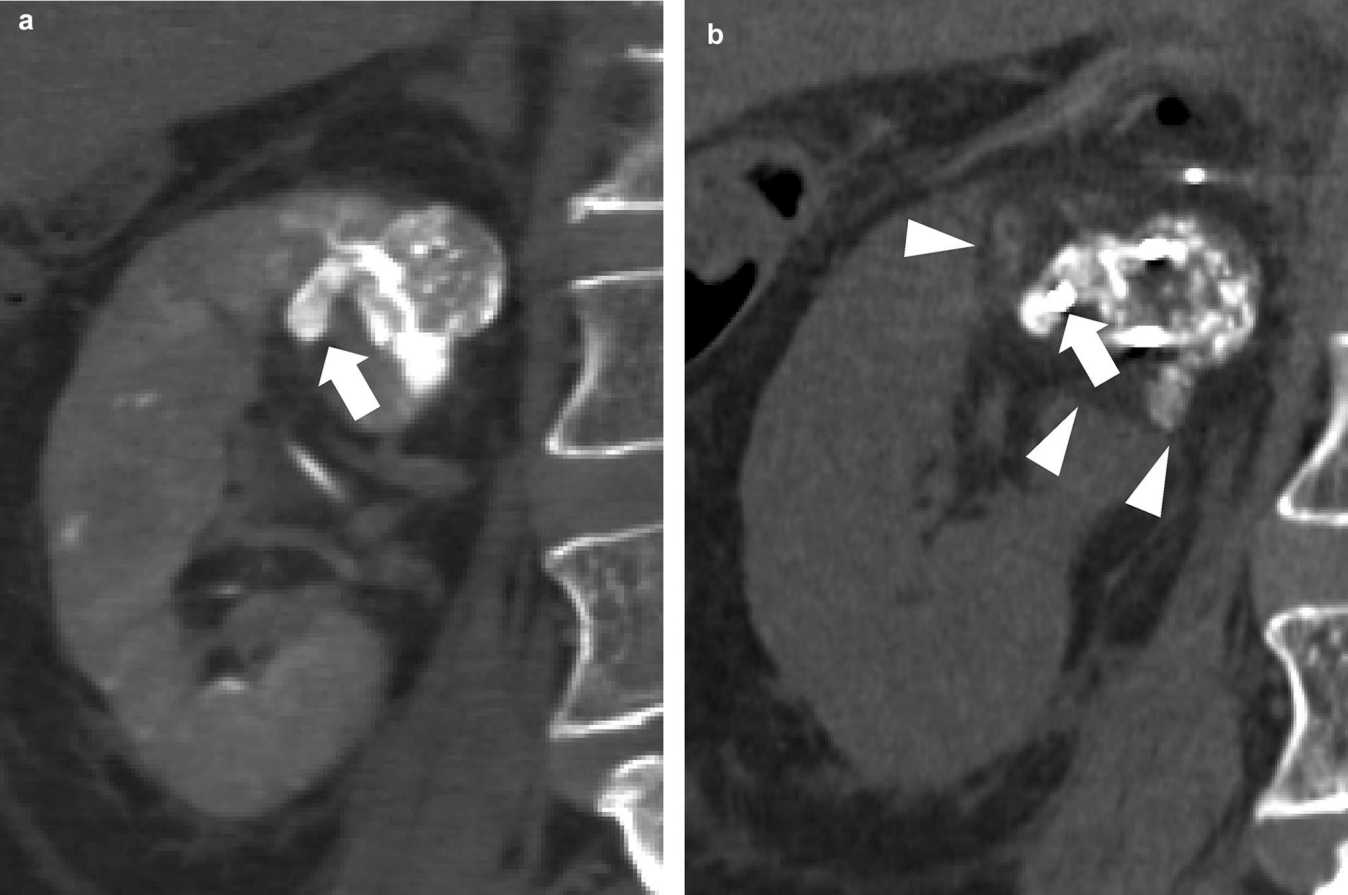
CT images of 2.9 cm T3a RCC in the right kidney in a 77 year-old man. **a** Coronal CT image immediately after TAE shows lipiodol accumulation in RCC, including the extension into the segmental vein (arrow). **b** Coronal CT image during ablation shows a cryoprobe directly inserted into the extension part into the segmental vein (arrow) and ice ball (arrowhead). This RCC achieves complete response. *CT* computed tomography; *RCC* renal cell carcinoma; *TAE* transcatheter arterial embolization

**Fig. 2:**
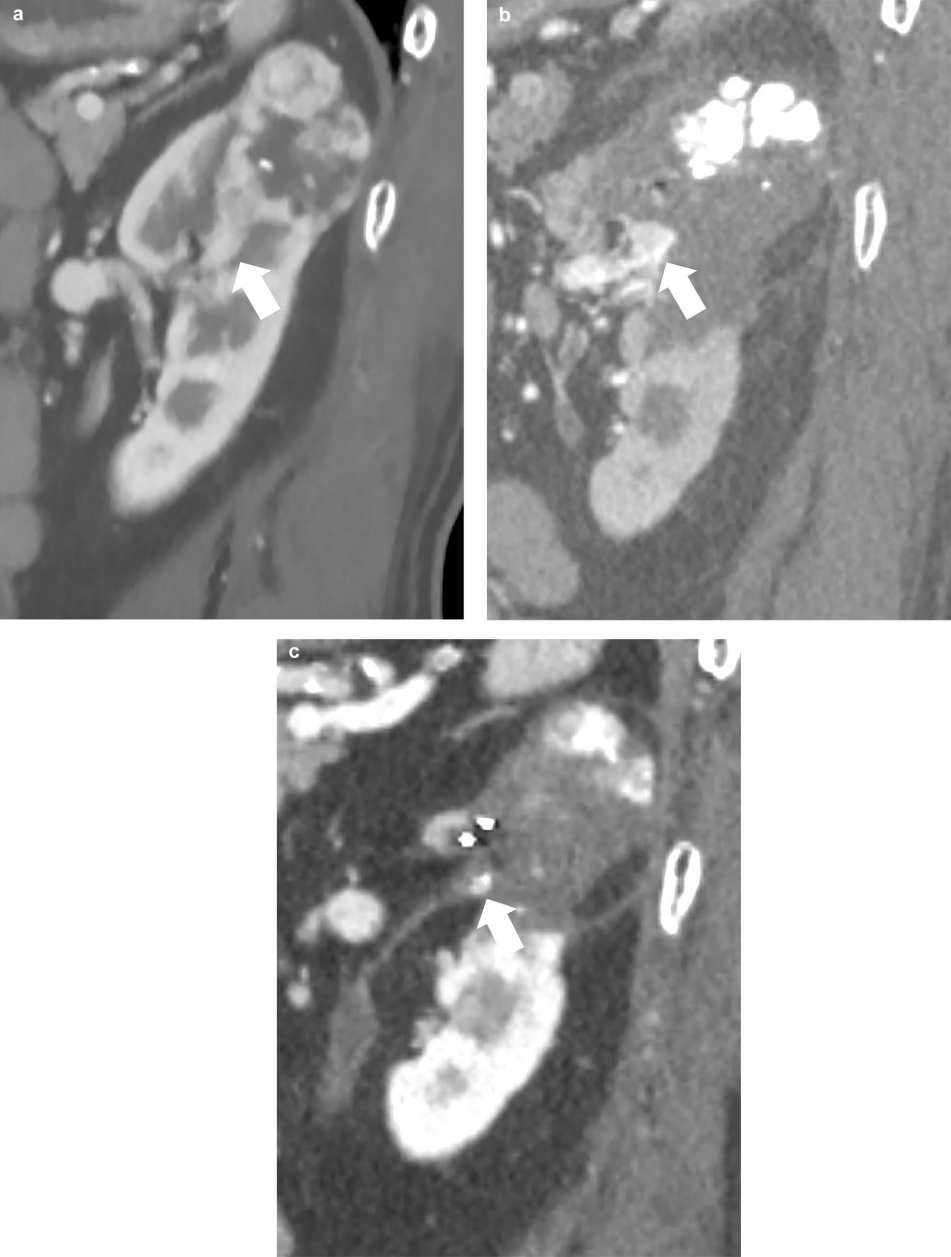
CT images of 4.4 cm T3a RCC in the left kidney in a 74 year-old man. **a** Sagittal CT image before treatment shows target RCC with the extension part into the segmental vein (arrow). **b** Sagittal dynamic CECT image (corticomedullary phase) 1 month after initial cryoablation shows the enhanced extension part into the segmental vein without lipiodol accumulation (arrow). Second cryoablation was performed 1.5 months after the initial cryoablation. **c** Sagittal CT image 51 months after initial cryoablation shows completely ablated T3a RCC with lipiodol accumulation (arrow) without enhancement. *CT* computed tomography; *CECT* contrast-enhanced CT; *RCC* renal cell carcinoma

Cryoablation was technically successful in all 14 T3a RCC cases (Figs. 1 and 2). Twelve RCCs underwent cryoablation once, and two underwent second cryoablation at 1.5 and 27 months after initial cryoablation, respectively, because of local tumor progression. In 14 of 16 treatments, TAE was performed 1−7 days (median, 2 days) before cryoablation, and in one case, TAE was performed 22 days before cryoablation (this was an early case and our protocol was not yet set, and the timing of this case fit the patient’s schedule). One patient underwent cryoablation without prior TAE. A median of three cryoprobes (2−5) was used to treat each tumor. The mean total ablation time was 51.6 ± 21.9 min (30−105 min). After 16 cryoablations, two minor AEs (one grade I hemothorax and one grade II hematuria) occurred. No major AE occurred.

All 14 patients were alive without distant metastases (e.g., lymph node, lung, and bone metastasis) with a median follow-up period of 45 months (6−93 months). A complete response was achieved by initial cryoablation in nine of 14 T3a RCCs (64.2%). Local tumor progression developed in five RCCs at a median of 12 months (1−21 months), and all had an intravenous extension. Two of the five patients with local progression after the first cryoablation had an indistinct border of the intravenous extension. However, the mean length of the intravenous extension was not significantly different between the local progression and non-local progression groups (10 mm and 8.3 mm, respectively) (*p* = 0.177).

**Fig. 3:**
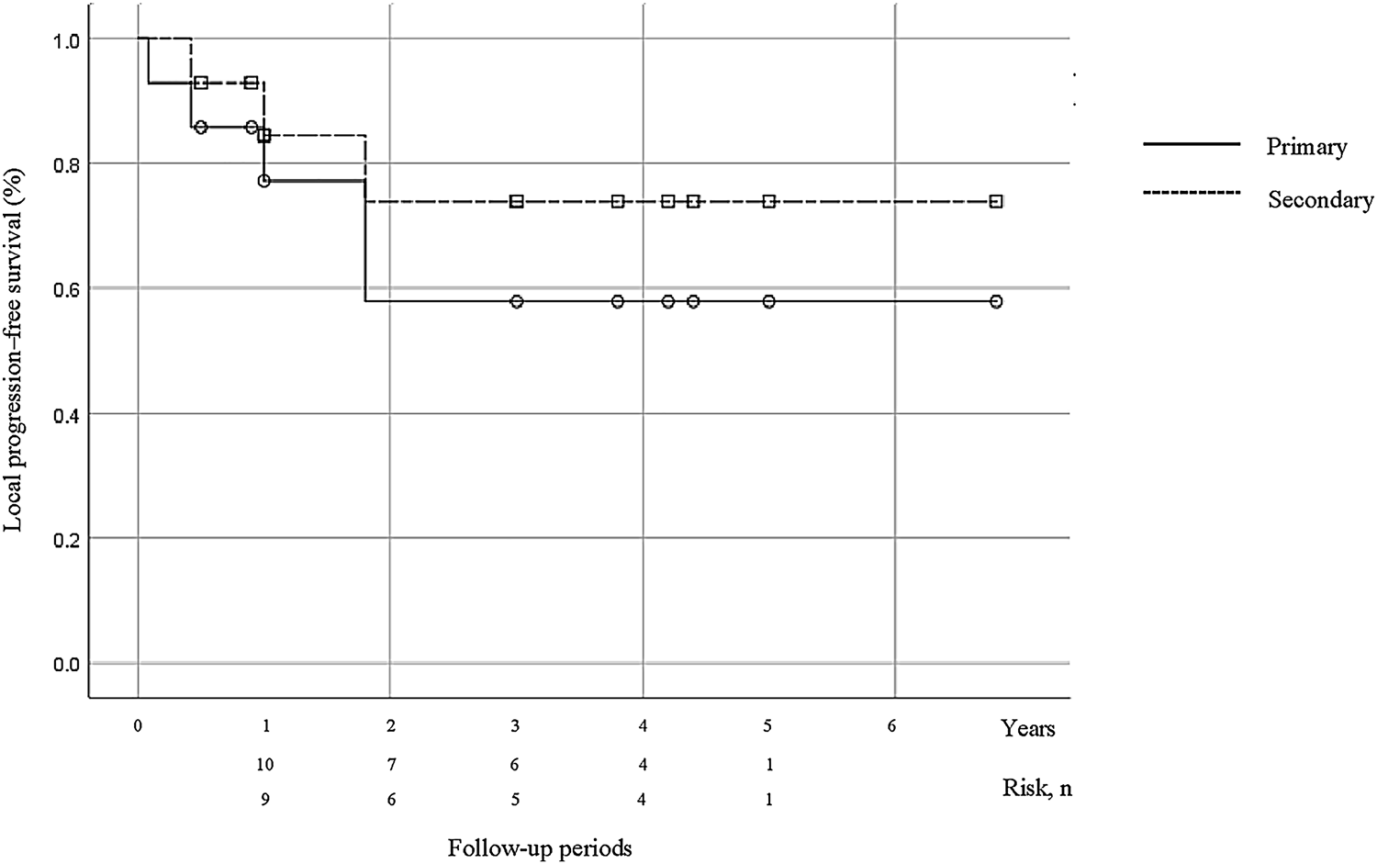
Kaplan–Meier curve demonstrates the technique efficacy rate of cryoablation for T3a RCC

Of the five cases with local tumor progression, two patients underwent secondary cryoablation. Thereafter, no local tumor progression developed. The other three patients underwent total nephrectomy 7, 16, and 46 months after cryoablation. All three cases had an intravenous extension, and two of them had an indistinct border of the intravenous extension. In total, in 11 of 14 T3a RCCs (78.6%), complete response was achieved by cryoablation alone. The primary and secondary technique efficacy rates were 77.1% and 84.4% at 1 year, 57.9% and 73.9% at 3 years, and 57.9% and 73.9% at 5 years, respectively (Fig. 3).

One patient underwent scheduled total nephrectomy for an 8 cm T3a RCC in the contralateral kidney 1 month after cryoablation and started dialysis after a total ipsilateral nephrectomy 46 months after initial cryoablation due to local progression.

The patient's eGFR values before and 6 months after cryoablation were 37.8 mL/min and 19.8 mL/min, respectively. In the other 12 patients, excluding the one on dialysis, the mean eGFR values before and 6 months after cryoablation were 63.4 ± 18.0 mL/min (37.9–88.3 mL/min) and 53.4 ± 18.0 mL/min (28.3–81.2 mL/min), respectively. A significant difference was found before and 6 months after cryoablation. (*p* < 0.001). In these 12 patients with a median of 41 months of follow-up (6–93 months), the final mean eGFR value was 50.9 mL/min (29.4–81.3 mL/min). None were on dialysis.

## Discussion

This study revealed that percutaneous cryoablation was safely performed with acceptable local control in selected patients with locally advanced RCC. The survival rates were high, and no major complications occurred. Our results may provide an alternative treatment option for non-surgical candidate patients with T3a N0 M0 RCC.

Although the accuracy of CT and MRI in the staging of RCC is usually high, the sensitivity and specificity may be lower in T3 tumors than in other stages. In the evaluation of preoperative CT of 96 patients with 100 pathologically proven RCCs, the sensitivity for the identification of peritumoral fat, sinus fat, and renal vein invasion was 77%, 86%, and 86%, respectively, and the specificity was 72%, 88%, and 97%, respectively. The sensitivity and specificity for the prediction of T3a tumors were 72% and 70%, respectively (*κ* score = 0.38 [0.29−0.47]) [14]. Thus, there might be other pT3a RCCs (diagnosed as clinical T1a by CT) treated by percutaneous cryoablation during the study period at our institution. However, Catalano et al. reported that axial and multiplanar reconstructed images of high-resolution CT scans with 1 mm slice are accurate in the preoperative evaluation of 40 patients with RCC. They were able to diagnose fat infiltration on 1 mm scans with 96% sensitivity, 93% specificity, and 95% accuracy [15]. We assessed target RCC using dynamic CECT images with ≤ 1.25 mm slice thickness and multiplanar reconstruction prior to treatment.

Radical nephrectomy is the standard treatment for T3a N0 M0 RCC. Survival may be expected if surgery can be performed. In a multicenter study with 861 pT3a RCCs, the median actuarial cause-specific survival of patients who underwent partial nephrectomy (*n* = 72) and radical nephrectomy (*n* = 789) was 7.7 years and 14.7 years, respectively [16]. Tumor size is an important factor for predicting the outcome of patients with T3a RCC. In patients with T3a RCC who were treated by surgical resection, a univariate analysis revealed that tumor size was significantly associated with disease-specific survival (hazard ratio: 1.09, 95% confidence interval: 1.05–1.12, *p* < 0.001) [17]. In T3a ≤ 7 cm N0 M0 (*n* = 205) and T3a > 7 cm N0 M0 RCC (*n* = 163) patients, the 5 year disease-specific survival rates were 76% and 65%, respectively [17]. All 14 RCCs were < 7 cm. If larger targets were included (i.e., > 7 cm), local tumor control and survival would have been lower and shorter, respectively.

In prospective studies, the 1-, 3-, and 5 year local effectiveness of percutaneous cryoablation for small RCCs (i.e., T1 RCC) exceeded 94% [18, 19]. In contrast, cryoablation for T3a RCCs may be more difficult and dangerous due to the heat sink effect and the risk of various complications, such as bleeding and collecting system injury. However, cryoablation may be preferable for centrally located renal lesions, including T3a RCC, compared to other heat-based ablation therapies, such as radiofrequency and microwave ablation, given that cryoablation may be less likely to injure the collecting system. In a retrospective review of 67 patients involving the ice ball overlapping the renal sinus, none of the cases were complicated by collecting system injury [20].

In a previous study of seven patients with T3a RCC, Atwell et al. reported high safety and effective local tumor control of percutaneous cryoablation, and, similar to our results, there was one major complication (hemothorax with chest tube placement). Furthermore, six of seven RCCs (86%) showed no local tumor progression with a median follow-up of 11 months [7]. Additionally, renal function was not affected. They commented that the reason for this low complication rate was unknown, but may be related to the precise cryoprobe placement afforded by image guidance, limiting trauma to the normal vasculature and parenchyma of the kidney. Prior TAE was performed in all 14 patients except for one. With this assistive procedure, target T3a RCCs were more clearly visualized on CT fluoroscopic images, allowing for more accurate puncture with a decreased risk of bleeding. In our study, we found that the invasive areas of tumors with intravenous extension were all segmental veins, and well-defined intravenous extension and excellent tumor control were observed by directly inserting a cryoprobe into the extension part of the RCC to the renal sinus. In contrast, tumors with indistinct borders are difficult to control using cryoablation.

In patients with T3a N0 M0 RCC, careful follow-up of the appearance of both local progression and distant metastasis is essential after treatment. Sameh et al. reported that the 2 year recurrence-free survival was 68% for pT3a N0 M0 in 57 patients who underwent surgery (mainly radical nephrectomy) [21]. Since distant metastases, such as lung, bone, and lymph node metastases, may appear in patients with T3a N0 M0 RCC, even if complete local control is achieved by percutaneous cryoablation, the outcome of cryoablation in long-term survival remains unknown.

This single-center retrospective study had several limitations. The study group was small, and follow-up was limited. The diagnosis of T3a RCC was made using dynamic CECT. Therefore, our 14 tumors were not pathologically staged. In addition, although percutaneous cryoablation showed acceptable local tumor control, our study was not designed to compare oncologic outcomes of T3a RCC patients treated with cryoablation and those treated with partial nephrectomy. In the future, to this end, it would be interesting to compare a larger number of such patients in the context of a multicenter randomized controlled trial.

In conclusion, percutaneous cryoablation was safe and effective in T3a RCC, which mainly involved the renal venous branches. This therapy may represent a viable option for certain selected patients with T3a RCC.

## Abbreviations

RCC: Renal cell carcinoma
CT: Computed tomography
MRI: Magnetic resonance imaging
CECT: Contrast-enhanced computed tomography
AE: Adverse event
TAE: Transcatheter arterial embolization
eGFR: Estimated glomerular filtration rate
